# A review of the effects of healthcare disparities on the experience and survival of ovarian cancer patients of different racial and ethnic backgrounds

**DOI:** 10.20517/2394-4722.2018.25

**Published:** 2019-02-27

**Authors:** Matthew Kaufman, Ana Cruz, Janese Thompson, Srinivasa Reddy, Nisha Bansal, Joshua G. Cohen, Yanyuan Wu, Jay Vadgama, Robin Farias-Eisner

**Affiliations:** 1 Obstetrics and Gynecology, University of California, Los Angeles, CA 90024, USA.; 2 School of Medicine, Meharry Medical College, Nashville, TN 37208, USA.; 3 Internal Medicine, Charles Drew University, Los Angeles, CA 90059, USA.

**Keywords:** Ovarian cancer, healthcare disparities, public health, racial disparities, epidemiology, literature review

## Abstract

Ovarian cancer (OC) is a serious condition that often presents at advanced stages and has high mortality rates, with the current mode of early-stage screening lacking sensitivity and specificity. OC often presents asymptomatically, which renders early diagnosis difficult. Furthermore, many patients lack significant risk factors or family history of the disease. Five-year survival rates differ between patients with OC among racial, ethnic, and social groups as a result of different social barriers. This review article aims to present the currently existing data regarding health care disparities among OC patients of different ethnic, demographic, and socioeconomic backgrounds, and what next steps should be taken to better understand and eventually eliminate these potentially devastating health care disparities. Increasing data support the notion that a combination of genomic, socioeconomic status, social factors, and cultural differences lead to differential treatments and therefore health care disparities. While genomic and biological factors are important, language barriers, geographic and travel barriers, differences in comorbidity likelihood between populations, and different treatment plans seem to have a greater impact on 5-year survival rates of patients from diverse backgrounds. Language barriers limit a shared-decision model of care. Transportation limitations and geographic differences can lead to limited follow-up and insufficient care in resource and equipment restrictive settings. Patients with these barriers also tend to have a higher incidence of comorbidities that raise the mortality rate of OC. Further research needs to explore effective solutions to bridge health care disparities and understand why they occur.

## INTRODUCTION

Ovarian cancer (OC) is the most lethal gynecologic malignancy and tends to develop as a result of atypical cells in the epithelium of the distal fallopian tubes^[1]^. Worldwide each year there are an estimated 200,000 women diagnosed with OC and 125,000 deaths from the disease^[2]^. In the United States, the American Cancer Society estimated that in 2017, there would be 22,440 new cases of OC and 14,080 deaths associated with the disease^[3]^. One in 78 American women will develop OC in their lifetime^[2]^. Five-year survival rates vastly differ among early stage and late-stage disease. For instance, with advanced stage III and IV disease, in which disease has spread beyond the pelvis to lymph nodes or the abdominal cavity or lungs, the mortality rate is around 70% whereas in early stages when the disease is confined to the ovaries or pelvis mortality rates are much lower at around 10%−40%^[1,4,5]^. Early diagnosis of OC when the disease is limited to the ovaries is difficult because, in early-stages, patients tend to be asymptomatic. This is further complicated by the lack of screening or diagnostic tests, which aid in early diagnosis^[1,4–6]^. Early diagnosis is prudent since the mortality rate after treatment or interventional therapy of early-stage OC is much lower^[1,5,6]^. By the time patients develop symptoms, such as abdominal pain or swelling, the vagueness of these symptoms often complicates and delays diagnosis. These symptoms tend to be attributed to aging, menopause, dietary changes, stress, depression or gastrointestinal issues. Patients with more apparent symptoms such as a pelvic mass, abdominal pain, bloating, abdominal swelling, early satiety or urinary symptoms often have a more advanced disease, with symptoms usually due to the development of large mass and ascites^[7]^. This is further complicated by the fact that screening tests for OC, such as tumor markers or imaging, often lack both sensitivity and specificity. As a result, a physician’s ability to detect OC in its early-stages is limited by the low-performance measures of these currently available screening tests^[5]^, leaving most patients undiagnosed. Patients with lack of access to tertiary medical care are more susceptible to late-stage diagnosis, associated with far higher mortality rates^[1,5,8]^. The current standard is to test for high levels of cancer antigen 125 (CA-125), an ovarian cell glycoprotein^[5]^. However, high levels of CA-125 tend to be correlated with late-stage OC^[5]^. Eighty percent of patients with late-stage OC have elevated values of CA-125, whereas only 10% of patients with early-stage OC have elevated values of CA-125^[5]^. Furthermore, certain histologic types of OC, such as mucinous or endometrioid type tumors may not have elevations in CA-125. Another factor limiting the utility of CA-125 screening is that high levels of CA-125 can be seen in a variety of other benign gynecologic and non-gynecologic malignant conditions. Non-malevolent conditions such as endometriosis, fibroids, and pregnancy can result in elevations in CA-125^[4]^. Other malignant conditions, which can result in elevations in CA-125, are breast, pancreatic, lung, gastric and colorectal cancers^[4]^. Additional diagnostic techniques such as ultrasound evaluation for masses, may aid in diagnostic precision but also lack sensitivity and specificity. Consideration of other risk factors for OC such as genetic susceptibility, strong family history, and nulliparity may influence the detection of OC in patients^[1,9]^. At present, the standard tests and techniques to screen for this devastating disease are ineffective, and studies show that routine screenings are not recommended for the general population^[4,8–10]^.

Standard treatment for OC is a combination of surgery and chemotherapy^[4]^. Surgical exploration is performed if there is sufficient suspicion for OC based on an initial evaluation. The goal of surgery is to confirm if malignancy is present and if so to proceed with surgical staging and cytoreduction. Per National Comprehensive Cancer Network (NCCN) guidelines, adjuvant therapy, which consists of platinum-taxane based combination chemotherapy, is necessary in most cases and depends on the stage of the disease^[5]^. Women with clinical suspicion of OC should be referred to a gynecologic oncologist for counseling and surgical treatment. Evidence shows that prognosis is improved when a gynecologic oncologist performs surgical staging and cytoreduction^[1,2,6]^. Unfortunately, despite initial therapy, the majority of women with advanced-stage OC will relapse and require additional treatment. The likelihood for recurrence depends on many factors, including distribution of disease at initial presentation, the success of initial surgical cytoreduction, rapidity of CA-125 resolution, and treatment response after primary therapy^[5]^. However, a predictive marker for recurrence has not been prospectively verified. Patients should be followed closely by a gynecologic oncologist to detect these signs of recurrence. The management of patients with relapsed disease varies based on the platinum-free interval or months since last platinum treatment. Platinum free interval has been shown to correlate with progression-free survival and overall survival, as well as response to subsequent treatment. The complexity of managing patients with relapsed disease underscores the importance of treatment plans to include a gynecologic oncologist. Mortality rates for advanced stage disease are around 70%, and these rates differ between patients with OC among racial, ethnic, and social groups could be a result of different social barriers^[1,4,5]^.

## EPIDEMIOLOGY OF OC

OC is the sixth most common cancer in women and the seventh most common cause of cancer death^[11]^. There is substantial geographic variation in OC incidence and mortality. Higher rates are seen in the United Kingdom, Northern Europe, Australia, and the USA, with lower rates observed in Asia, China, and Africa^[11]^. A recent study examining the international assessment of OC incidence and mortality confirmed these findings, demonstrating the lowest incidence of OC in China, and the highest in Russia and the United Kingdom^[12]^. Similarly, other studies have confirmed that countries with a predominantly Caucasian population such as Europe, the US, Canada, and Australia, have a higher incidence of OC and that the incidence is lower in countries with other ethnic groups such as Asia, Brazil, and Mexico^[12]^. These findings are consistent with the fact that within the USA, rates of OC are higher among white women than black women^[11]^. Incidence rates of OC in the US are also lower in Asian/Pacific Islanders, American Indians/Alaskan Natives that are also consistent with previous international studies. In the US the incidence rate per 100,000 women of OC is 13.5 in whites compared to 9.9 in Asian/Pacific Islanders, 10.6 in American Indians/Alaska Natives, and 10.0 in blacks and 11.6 in Hispanics^[12]^. In other terms, compared to black and Hispanic women, the risk of OC is 40% greater in white women^[2]^. The exact reason for this racial disparity in incidence is unknown however racial disparities in certain risk factors for OC may account for this variation. For instance, this variation in geographic incidence rates of OC may be attributed to genetic risk factors, such as the higher incidence of OC among women with Ashkenazi Jewish ancestry^[13]^. Among women with OC with Ashkenazi Jewish ancestry, 40% have a mutation in *BRCA1* or *2* when diagnosed with OC^[2]^. Other risk factors for OC such as obesity, endogenous or exogenous hormonal exposure, parity, and dietary factors such as smoking, alcohol use, caffeine consumption are highly related to cultural habits and lifestyle practices that differ across different ethnic groups and may account for this geographical variation^[2,12]^. Racial discrepancies of rates on gynecologic surgery such as tubal ligation and hysterectomy, which are known protective factors against OC, may also account for some of these variations in OC incidence^[2]^. Similarly, rates of breastfeeding and combined oral contraceptive pill use, also known protective factors against OC, are highly related to cultural practices and may differ across ethnic groups. Overall the incidence of OC has gradually declined in most developed countries such as North America and Europe since the 1990’s^[14]^. Conversely, less developed countries with recent economic growth and lifestyle changes have seen increases in incidence rates^[14]^.

## DISPARITIES IN THE SURVIVAL AND EXPERIENCE OF OC PATIENTS

The overall difficulty of early detection, early diagnosis, and the subsequent optimum treatment for patients with OC is exacerbated by the social disparities that exist in underserved communities. These disparities, in turn, lead to differences in survival rates and treatment. The existing literature supports the hypothesis that the risk of all-cause mortality in African American OC patients is roughly 1.3 times higher when compared to Caucasian women with OC, even when the access to care is equal^[15]^. The mortality rates have increased specifically for African American populations over time^[15,16]^. A study conducted by Srivastava *et al.*^[16]^ demonstrated that from 1992 to 2008 the 5-year survival rates for Caucasian women rose from 40.7% to 45%, yet 5-year survival rates for African American women declined over that same time period from 47.9% to 40.3%. A similar study found that the 5-year survival rate for African American women had fallen to 36% from 2006 to 2012, while all races combined had a 45% 5-year survival rate, with Caucasian women having the highest 5-year survival rate at 46%^[16]^. This variation in survival rates appears to be due to a decreased likelihood of receiving guideline-recommended care^[17]^. Other studies have suggested that after controlling for factors like socioeconomic status (SES), patients with the same stage of OC have similar survival rates^[3,18]^. Studies suggest that patients of a lower SES are receiving care less in line with NCCN guidelines and have decreased access to preventative medicine and genetic testing, which could contribute to delayed presentation. However, most studies are limited by their inability to detect if a patient’s SES and a delay of presentation to the clinic are correlated, which could, in turn, affect 5-year survival rates^[3,18]^. The current literature on the different survival rates of OC between diverse populations and the frightening statistics facing our underserved communities introduces a series of compelling questions. Why are patients with low SES presenting to the clinics with a more advanced disease? What preventative factors (i.e., the difference in the fund of knowledge, access, communication and financial resources) impact these differences in survival? Are there certain factors that are creating more disadvantages for patients who are underserved than other factors? What can be done to effectively mitigate these disparities? Studies have tried to control for such factors, with little success in large part because a multitude of critical contributing confounding variables is at work, not just one sole factor that is key in precluding underserved communities from attaining equal outcomes^[18,19]^. Currently there is a need for increased randomized control studies and interventional studies that could shed more light on the impact of these social determinants of health, however the challenge is to isolate certain variables in these studies and have a sample size large enough to draw impactful conclusions.

## GENOMIC DISPARITIES AMONG OC PATIENTS

The lifetime risk of developing OC is low, at 1.5%, and the vast majority of patients who develop OC comprise sporadic cases with no significant family history of the disease^[1]^. In contrast, the remaining group of patients with deleterious genomic germline mutations (i.e., *BRCA1* and *BRCA2* genes) carry a much greater lifetime risk for breast cancer and OC (i.e., 50%−85% and 20%−40%, respectively)^[1,20–23]^. Patients with a known genetic susceptibility to OC are typically followed closely with a combination of pelvic examination, ultrasound imaging, and CA-125 testing in order to detect abnormalities at early curable stages. These patients are also typically offered risk-reducing surgery, which consists of removal or the fallopian tubes and ovaries at the completion of childbearing in order to prevent the onset of disease. Patients in underserved communities may have lack of access to this type of preventive medical care. Among these OC patients with deleterious germline mutations, a significantly decreased overall survival and the worse prognosis is reported in OC patients from underserved populations^[24–26]^. Bandera *et al.*^[27]^ reported an increased OC risk, associated with a worse survival rate, in underserved communities after controlling for confounding variable. Factors, such as medical comorbidity risk, and prevalence of *BRCA1* and *BRCA2* mutation, pointing instead to other contributing etiologic factors, such as quality of care and delay in care, to explain the significantly poorer outcome. Although a higher incidence of unmanaged medical comorbidities (i.e., hypertension, coronary artery disease, and diabetes) may be associated with an increased OC risk, these medical comorbidities alone cannot explain the poor outcomes in underserved populations^[16,27–29]^.

## PUBLIC HEALTH, SOCIAL DISPARITIES, AND LANGUAGE BARRIERS AFFECT OUTCOMES IN OC PATIENTS

High adiposity and inflammatory diets consisting of high sugar intake are associated with an increased risk of developing OC^[16,27–30]^. A systematic review of 28 studies reported a statistically significant, association between obesity [body mass index (BMI) 30 kg/m^2^ or more] and OC^[31]^. Another large prospective study found that the risk of death from OC was higher in women with the greatest BMI (35–40 kg/m^2^) compared with those of normal weight (BMI 18.5–24.9 kg/m^2^)^[32]^. African American and Hispanic women have a disproportionately higher rate of obesity and have higher BMI (> 40) which may correlate with a higher incidence mortality of OC^[16,27,28,33]^. A correlation also exists between obesity and socioeconomic position, suggesting that more overweight and obese people are from underserved populations with poorer access to healthcare^[34]^. In addition to limited access to quality healthcare, obese patients may be more difficult to diagnose with OC due to greater difficulty in assessing vague symptoms and limited utility of current diagnostic approaches in these patients. Studies indicate that women who were considered overweight and obese had symptoms such as abdominal swelling and discomfort months before the diagnosis of OC^[35]^. Healthcare providers can impact meaningful change through education to patients of the positive impact of a low inflammation diet. Similarly, patient education regarding weight loss through healthy diet and exercise can meaningfully impact patient’s health and reduce risk factors for OC.

Patients with a limited English proficiency have lower health literacy rates, less access to healthcare systems, and poorer outcomes^[36]^. Many of these patients use ad-hoc medical interpreters, such as staff, friends or family members, who are not officially licensed to serve as an interpreter for a clinical interaction. The use of an ad-hoc interpreter leads to miscommunication and is ineffective and result in poor patient outcomes. One study confirmed that the use of an ad-hoc interpreter leads to a higher rate of error than using no interpreter at all^[37]^. Schwei *et al.*^[36]^ reported that patients rarely, if ever, request interpreter services. Even the use of trained medical interpreters may result in impaired physician-patient communication. Kamara *et al.*^[38]^ analyzed 24 audio recordings of cancer genetic counseling consultations from 13 different patients and six different counselors conducted in Spanish with the assistance of 18 telephone interpreters at two large public hospitals^[38,39]^. The primary causes of impaired communication through the use of interpreters were erroneous hypothetical explanations, and misinterpretation by the interpreter, resulting in a significant departure from the shared-decision model of healthcare^[37,38,40]^. The interpretations did not assist patients in their decision-making process. Furthermore, physicians found that even with the use of interpreters it was difficult to understand the decision of a patient. There were instances in which interpreters did not interpret a patient’s response or question, a marked departure from a high-quality mutual discussion, and a shared-decision model of healthcare^[36–38,40]^. Therefore, patients often consented to the provider’s recommendations regardless of their comprehension of the treatment plan and limiting patient autonomy. Low English proficiency patients are more likely to have a limited health literacy and increased dissatisfaction with the health system overall^[36–38,40]^. These data suggest that public health, social disparities, and language barriers negatively impact outcomes in OC patients.

## GEOGRAPHIC AND TRAVEL FACTORS THAT IMPACT OC PATIENTS

Disparities also exist in patients’ geographic environments, impacting patient outcomes. Patients from lower socioeconomic communities have access to fewer clinics, with fewer resources and compromised diagnostic capability^[19,29]^. Sakhuja *et al*.^[19]^ reported that African American OC patients lived in geographic areas that had fewer oncology centers and fewer centers with ultrasound, and fewer subspecialists^[1,5,19,29]^. Statistics have consistently shown that patients with OC are best treated by a gynecologic oncologist and that tertiary care medical centers have the resources and capabilities to better serve these patients. Lack of access to subspecialty care negatively impacts survival from OC. Hence, the likelihood of a patient presenting to a physician with insufficient clinic resources is statistically much greater in a lower socioeconomic setting. Moreover, OC patients who are presented to general hospital clinics have greater odds of late-stage diagnoses and increased mortality rates than those who present to subspecialty clinics^[18,19]^.

Srivastava *et al.*^[16]^ reported that only 22% of Caucasian and 14% of African American OC patients would be willing to drive more than 20 miles for treatment, which also correlates with data suggesting that the larger the distance between a patient and their gynecologist, the greater the mortality rates^[16,41,42]^. Guidry *et al*.^[43]^ surveyed 593 cancer patients in Texas, of which 38% of the Caucasians surveyed, 55% of the African Americans, and 60% of the Hispanic respondents reported that they had poor access to a vehicle^[16,41,43]^. The net result to the underserved communities was geographic and transportation barriers, greater reschedules, no-shows, delayed care or missed or delayed medication use^[16,41]^. Hence, these geographic and transportation factors lead to delivery of substandard care and negatively, impact survival rates^[19,29,41]^. Across 25 different studies, patients reported that transportation was a major barrier to their healthcare^[41]^. These data indicate that geographic and travel factors have a negative impact on OC patient outcome.

## DIFFERENT OC PATIENTS RECEIVE DIFFERENT TREATMENT

Patients from different socioeconomic backgrounds and cultures experience a difference in quality treatment. African American women from underserved communities overall have lower 5-year survival rates from OC than their Caucasian counterparts, attributed to treatment delays^[16]^. Bristow *et al.*^[44]^ reported that despite equitable access to healthcare, patients who hailed from lower SES status were less likely to receive treatment adhering to the NCCN guidelines^[29]^. Furthermore, a clear difference exists between populations regarding access to quality of healthcare, impacting the outcome of an OC patient. To understand the source of healthcare disparities is the key in order to pursue strategies to correct these disparities and eventually create initiatives to eliminate them.

An increasing body of literature suggests that patients from a lower SES receive substandard care, in large part from a general lack of proper facilities and equipment to carry out standard procedures^[1,16,29,44]^. Unfortunately, patients from underserved communities, have poorer access than affluent communities to high-quality healthcare^[19,29]^. African American and Hispanic patients were less likely to receive accurate staging for OC than Caucasian patients^[29]^. African American patients with OC also tend to receive less proactive treatment plans than Caucasian patients^[16,19]^. A study conducted at a large, high volume medical center showed that African American OC patients were less likely to undergo surgery than Caucasian OC patients overall, 61% and 77% respectively, and less likely to undergo chemotherapy^[16,19,29]^. Furthermore, underserved patients are much less likely to enroll in clinical trials and experimental treatments. In a study that analyzed over 400 clinical trials from The Gynecologic Oncology Group (GOG) from 1985 to 2013, 83% of participants were Caucasian, and only 8% African American, and 2.2% Hispanic^[29,42,45]^. As compared with the expected Centers for Disease Control projections for minority participation in clinical trials, actual African American participation was 15 times lower than expected for ovarian GOG trials^[45]^. The GOG also reported that the rate of participation for African American patients has been decreasing over time, demonstrating that participation from African American communities has decreased 2.8 times from 1994–2002 to 2009–2013^[45]^. There are many possible explanations for poor participation by minority groups in clinical trials include lack of access to centers, which offer these trials, lack of understanding regarding the purpose and potential benefits of these trials, and lack of means such as transportation to effectively participate in these trials. Due to decreased levels of education, patients may have diminished understanding of how the treatment works and therefore cannot understand how these treatments are different from previous ones and might have decreased efficacy. Additionally, cultural and spiritual differences in these communities could also contribute to the disparity. Members of these communities have been spurned by healthcare institutions and other institutions in the past, which could result in an overall distrust in providers or healthcare institutions. All of these aspects stack the odds against minority communities from receiving proper treatment.

Armstrong *et al.*^[46]^ reported that only 37% of patients received genetic counseling prior to testing for the *BRCA1*/*2* gene, and of those that did receive counseling, most were Caucasian women (69%) who were married (76%), with high income (55%) and with a college degree (81.4%)^[1,46]^. African American women have a much lower rate of testing particularly in limited-resource settings, which is important because they have a similar or higher incidence of *BRCA* mutation than their Caucasian counterparts^[1,20–23]^. It is estimated that roughly 12.4% of African American patients with breast cancer under 50 carry the mutation^[1]^. Limited resource settings are less likely to have a genetic counselor, and therefore patients are less likely to receive counseling support. All patients with OC should receive genetic counseling according to the NCCN guidelines. By undergoing genetic testing and genetic counseling, care providers can have a better understanding of the type of tumor that a patient is presenting with^[21,38]^. If the patient has a known *BRCA1* or *2* mutation, then a provider has increased indications of what targeted therapies may work^[1,20–23,26]^. A *BRCA1* or *2* mutation may result in errors with homologous recombination mechanisms in the DNA repair system, consequently there are downstream genes that are more likely to be mutated as well. These downstream genes have been studied extensively and as a result, target-therapies have been developed targeting these mutations. Additionally, patients who receive genetic counseling and genetic testing have increased understanding of their cancer, which can help make more informed decisions when it comes to their care^[21,38]^. They have increased understanding about the progression of disease, their risks of developing cancer and their risks of passing this mutation onto their offspring. By providing this information, the decision-making model is shifting from one of paternalism to a shared-decision model, and allows for patients to be much more cognizant of early signs of developing cancer to begin treatment earlier, engage in more preventative treatment measures, and make care decisions in a timely manner that are in line with their values^[1,38]^. However, in this case patients with decreased SES and therefore decreased access to genetic testing and counseling providing care teams have less information on base treatment, which essentially limits patient involvement in their own care^[1,19]^. Proper genetic counseling prior to testing is a critical component of delivering this test and understanding the results.

Peres *et al.*^[47]^ reported that women taking an aspirin regimen for cardiovascular diseases or a daily nonsteroidal anti-inflammatory drug for arthritis had a decreased risk of developing epithelial OC, which stood at 44%, and 26%, respectively. However, a significant disparity still exists between the compliance rates for Caucasian and African American populations (44% *vs*. 29%, respectively) for these regimens^[48]^. Decreased compliance with these medications reduces the protective factor of these agents against OC. African American and Hispanic women are much less likely to use oral contraceptives, a known reducer of OC risk^[1,49]^. These data indicate that patients from underserved communities receive less preventive care than other patients.

## CONCLUSION

Eliminating healthcare disparities is critical in order to ensure optimal outcomes in all patients with OC. Identifying what the healthcare disparities are is critical to their elimination. A paradigm shift, which leads to redistribution in the allocation of healthcare resources to create more equality across populations, will eliminate healthcare disparities.

Future research must focus upon the underlying genetic components that contribute to healthcare outcomes. Research that will elucidate tumor and population-specific molecular modifications to genes and proteins may positively impact the outcomes of patients with OC. The contribution of changes in dietary considerations (i.e., low sugar), language barriers, and geographic differences to the elimination of healthcare disparities requires additional research.

Physicians can impact the elimination of healthcare disparities through patient education (i.e., dietary practices), effective use of interpreters, and outreach to resource-poor communities with less access to high-quality healthcare. Population data demonstrate that the allocation of important equipment and resources (i.e., ultrasound machines, special genetic counselors) to support community primary care physicians, and the number of offers obstetric and gynecological specialists in the community will favor a lower incidence of late-stage diagnosis of OC^[19]^. Patients hailing from lower SES and underserved communities may be at an additional disadvantage when they are excluded from promising investigational clinical trials. Hence, the concept of the mobile clinic may bring high-level care directly to communities obviating the need for patients to travel long distances to find high-quality healthcare^[19]^.

In the short-term and long-term, a robust and sustainable solution is the creation of an inclusive culture that supports the elimination of healthcare disparities and discrimination among different racial, ethnic and social groups^[50]^. The net result would be the replacement of patient isolation in underserved communities with access to quality healthcare, including access to promising clinical trials to treat cancer.
